# Genome analysis of DNA repair genes in the alpha proteobacterium *Caulobacter crescentus*

**DOI:** 10.1186/1471-2180-7-17

**Published:** 2007-03-12

**Authors:** Marinalva Martins-Pinheiro, Regina CP Marques, Carlos FM Menck

**Affiliations:** 1 Department of Microbiology, Institute of Biomedical Sciences, Av. Prof. Lineu Prestes 1374, São Paulo, 05508-900, SP, Brazil

## Abstract

**Background:**

The integrity of DNA molecules is fundamental for maintaining life. The DNA repair proteins protect organisms against genetic damage, by removal of DNA lesions or helping to tolerate them. DNA repair genes are best known from the gamma-proteobacterium *Escherichia coli*, which is the most understood bacterial model. However, genome sequencing raises questions regarding uniformity and ubiquity of these DNA repair genes and pathways, reinforcing the need for identifying genes and proteins, which may respond to DNA damage in other bacteria.

**Results:**

In this study, we employed a bioinformatic approach, to analyse and describe the open reading frames potentially related to DNA repair from the genome of the alpha-proteobacterium *Caulobacter crescentus*. This was performed by comparison with known DNA repair related genes found in public databases. As expected, although *C. crescentus *and *E. coli *bacteria belong to separate phylogenetic groups, many of their DNA repair genes are very similar. However, some important DNA repair genes are absent in the *C. crescentus *genome and other interesting functionally related gene duplications are present, which do not occur in *E. coli*. These include DNA ligases, exonuclease III (*xthA*), endonuclease III (*nth*), O_6_-methylguanine-DNA methyltransferase (*ada *gene), photolyase-like genes, and uracil-DNA-glycosylases. On the other hand, the genes *imuA *and *imuB*, which are involved in DNA damage induced mutagenesis, have recently been described in *C. crescentus*, but are absent in *E. coli*. Particularly interesting are the potential atypical phylogeny of one of the photolyase genes in alpha-proteobacteria, indicating an origin by horizontal transfer, and the duplication of the Ada orthologs, which have diverse structural configurations, including one that is still unique for *C. crescentus*.

**Conclusion:**

The absence and the presence of certain genes are discussed and predictions are made considering the particular aspects of the *C. crescentus *among other known DNA repair pathways. The observed differences enlarge what is known for DNA repair in the Bacterial world, and provide a useful framework for further experimental studies in this organism.

## Background

The removal of lesions from the DNA molecule depends, mostly, on cellular DNA repair capacity. Several highly conserved mechanisms efficiently remove those DNA lesions that frequently occur in the cell's genetic material, thereby ensuring genomic integrity. Most of what is known for bacterial DNA repair mechanisms derives from studies in *E. coli*. However, genome sequencing has revealed a large number of genes with unknown functions, and clear differences raise questions on the ubiquity of similar DNA repair pathways within the bacterial kingdom. For example, it was recently described for *Mycobacterium tuberculosis *that the induction of functional DNA repair genes by DNA lesions is not entirely dependent on RecA protein. In fact, there are at least two induction pathways for DNA repair genes in this gram-positive bacterium [1].

Alpha-proteobacteria include soil bacteria such as those involved in the global nitrogen cycle (*Bradyrhizobium*, *Mesorhizobium*, *Nitrobacter*, *Sinorhizobium *and *Rhizobium*), plant tumor-inducing bacterium (*Agrobacterium*), and bacteria frequently found in water (*Candidatus*, *Caulobacter*, *Erythrobacter*, *Jannaschia, Sphingopyxis*, *Silicibacter*). In this group of bacteria, pathogenic organisms are also included (*Anaplasma, Bartonella*, *Brucella*, some species of *Wolbachia*, *Rickettsia *and *Ehrlichia*). Other members present environmental interest as ethanol producers (*Zymomonas*), or by their capacity for degrading a wide range of toxic organic compounds, thereby assuming a bioremediation role (*Novosphingobium*, *Rhodopseudomonas *and *Rhodobacter*). The species *Gluconobacter oxydans*, by being able to oxidize a wide variety of substrates, has a great importance within the food and pharmaceutical industries [2,3].

The aquatic bacterium *Caulobacter crescentus *is an important model organism for studies on bacterial cell cycle and differentiation, but as for other alpha-proteobacteria, very little is known about its DNA repair pathways. *C. crescentus *has the ability to survive in low-nutrient environments, and produces two different cell types (a sessile stalked cell and a motile swarmer cell) which are, in fact, important life forms of its cell cycle [4]. Its genome, composed of 4,016,942 base pairs, encodes 3,767 genes [5]. The availability of the complete genome sequence from *C. crescentus *allows for an "in silico" analysis which may contribute towards understanding the strategies this bacterium uses to live, including how it processes its DNA molecule.

For DNA repair studies, photoreactivation was established in alpha-proteobacteria when *C. crescentus *[6] and *Rhodobacter *[7] bacteria were irradiated with ultraviolet (UV), and photorepair was observed when submitted to high doses of visible light. In both bacteria, genes that code for proteins of the photolyase family may perform this function. An *alk*B mutant in *C. crescentus *was shown as highly sensitive to the alkylating agent methyl methanesulfonate (MMS), and the expression of this gene is dependent on the cell cycle of the bacteria [8]. Additionally, DNA polymorphism at the *alkB *locus was previously described in *Brucella abortus *[9]. Recently, this gene has been described as related to a new DNA repair pathway, oxidative demethylation [10]. The description of a constitutive O_6_-methylguanine-DNA methyltransferase in *Rhizobium meliloti *confirms the presence of a third DNA damage reversal mechanism in the alpha-proteobacteria group [11].

The reactivation of bacteriophages in *C. crescentus *exposed to 5–10 J/m^2 ^of UV [6], the phenotypic characterization of mutants for *uvrA, uvrB *and *uvrC *in order to investigate the sensitivity to radiation in *Rhodobacter sphaeroides *[12], and functional complementation studies in *Sinorhizobium meliloti *[13] indicate the existence of an SOS response in these alpha-proteobacteria. This is confirmed by the identification of the *rec*A and *lex*A orthologs, which control the SOS regulon. In addition, several other studies have been carried out to unravel the SOS box in this group of bacteria [14-18]. Recent studies demonstrate the importance of DNA repair pathways in heavy metal stress in *C. crescentus *[19]. The phenotypes of mutants in the *xthA *gene of *Brucella abortus *[20] correlate well with the function of this gene in base excision repair (BER), as already described in *E. coli*. Finally, Galhardo *et al *[21] characterized the *imuAB dnaE2 *operon, indicating that it is involved in DNA damage induced mutagenesis under SOS regulon control in *C. crescentus*. However, in the same work, there was no evidence that another translesion DNA polymerase, DinB, was controlled by the SOS response in this bacterium, raising the possibility that a different regulatory pathway may be operational for this gene in this bacterium.

Here we present a comparison of the main DNA repair genes between *C. crescentus *and *E. coli*. For this analysis, we searched for open reading frames (ORFs) in the *C. crescentus *genome that present a significant similarity with known DNA repair related genes, especially those from *E. coli*. As the genes involved in DNA repair are, in general, part of the cell core metabolism, they maintain a strong similarity in different bacterial genomes, but intriguing differences suggest biological diversity in bacterial responses to DNA damage. This "in silico" study provides important insights regarding DNA repair pathways in *C. crescentus*, and those of the alpha-proteobacteria group, thereby giving a direction for further functional characterization.

## Results and discussion

The genome of *C. crescentus *was screened for the presence of genes known to act in the metabolism of DNA lesions, most in *E. coli*. This comparison lead to the identification of many of the DNA repair related pathways. In order to facilitate comprehension of the main similarities and differences among these genomes, genes have been classified as: **1-Excision repair **(base excision repair, nucleotide excision repair and mismatch repair); **2- Direct repair **(photoreactivation, alkyltransfer and oxidative demethylation); **3- Recombinational repair **and **4- Other DNA repair related proteins**.

### 1 – Excision repair

#### A – Base excision repair (BER)

The BER pathway has been found in all living organisms, and involves several different proteins with the functions of DNA glycosylases and endonucleases. These enzymes are able to remove damaged bases induced by different means, such as those lesions spontaneously generated by oxidative stress. In BER, damaged bases are removed from the DNA backbone in a free form by specific DNA glycosylases, leaving an abasic apurinic or pyrimidinic (AP) site. An AP-endonuclease nicks the DNA at the position of these abasic sites, and subsequently a repair patch is synthesized, thus restoring the original molecule [22]. The main genes involved in BER are listed in Table 1. There are two main types of DNA glycosylases, the Fpg/Nei and Nth/MutY families, both represented in the genome of *C. crescentus*. In addition to their glycosylase activity, several of these enzymes also display a lyase activity that cleaves the phosphodiester backbone 3' to the AP site, so they are known as bifunctional glycosylases. Although a Nei ortholog was not found, the Fpg protein may participate in the removal of oxidative lesions in this bacterium. The exonuclease III, coded by the *xth*A gene, is responsible for most AP-endonuclease activity in the BER system in *E. coli*. In *C. crescentus*, we found two distinct close orthologs of exonuclease III (*xthA*1, *xthA*2), but this does not necessarily represent redundancy. The second ortholog of XthAmay compensate for the absence of the endonucleases Nfo and Nfi. The gene for the other protein of the same family, *mutY*, is also found in one copy.

The *ung*/*udg *gene, which encodes uracil-DNA glycosylase, is critical for the removal of uracil from DNA. Uracil, which is normally misincorporated during replication, may also result from cytosine deamination, yielding C to T transition mutations [23]. The presence of four genes coding for proteins of the uracil-DNA-glycosylase family in *C. crescentus *suggests the importance of deamination of cytosine in this genome. Although they have no significant sequence similarity to the UDG protein found in *E. coli*, they all belong to the UDG family (COG1573) previously described in *Thermotoga maritima *[24]. Thus, these proteins may be important for the stability of this G:C rich genome [25]. Orthologs of the AlkA and Tag, which are the main proteins responsible for the repair of methylated bases in *E. coli *by BER, are also found in *C. crescentus *genome.

#### B – Nucleotide excision repair (NER)

Distortion in the double helix caused by certain lesions seems to be the first signal for the recognition of damage by the NER system, which is capable of recognizing a larger variety of base modifications, using more generic endonucleases than the BER system. Lesion removal in the intact oligonucleotide form, instead of as free bases as seen in BER pathway, is performed by the sequential action of damage recognition proteins, nuclease and helicase proteins, followed by DNA polymerization and ligation by DNA ligase [26]. This pathway, which mainly consists of the proteins UvrA, UvrB, the nuclease UvrC, the helicase UvrD and the dsDNA translocase Mfd, is complete in *C. crescentus *(Table 2).

**Figure 1:**
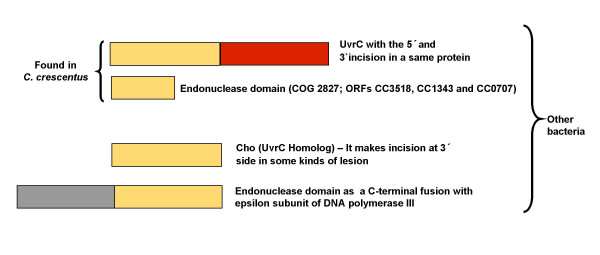
Scheme for the UvrC and UvrC-like proteins. The yellow boxes characterize putative endonuclease domains. In red box is shown the C-terminal domain of UvrC, and in gray box the epsilon subunit of DNA polymerase III.

It is interesting to note that in *C. crescentus *there are three copies of small genes corresponding to an endonuclease domain (COG2827). This domain [27] is also observed in the N-terminal portion of the UvrC protein, and the genes are often annotated in the genomes of several other organisms such as "UvrC-like protein, N-terminal". Although the role of these potential endonucleases in the repair of *C. crescentus *is unknown, other proteins, also containing a similar domain, have likewise been observed, as is the case of the protein encoded by *cho *(uvr**C ho**molog) gene of *E. coli*. Cho protein is a homolog to the N-terminus of UvrC, and makes a DNA incision at 3' from certain types of DNA damage, where UvrC is less efficient [28]. Cho is a damage inducible protein whereas UvrC is not, both being found together only in few bacteria [29]. Some of the Cho homologs, particularly those belonging to the beta proteobacteria group (Table 3), present an N-terminus fusion with a putative 3'exonuclease domain, similar to the epsilon subunit of DNA polymerase III. These two activities (endonuclease and exonuclease) of this fusion protein were proposed to act in coordinate and sequential functions in a new DNA repair mechanism [30]. These *C. crescentus *UvrC-like endonucleases encode proteins with sizes ranging from 96 to 123 amino acid residues, and are widespread in proteobacteria of the alpha group, although they can also be found in other bacterial groups (Table 3). Although these paralogs may indicate functional redundancy, they may play roles similar to those of the Cho protein. In other words, they may act in NER, but on different types of DNA damage, being back up enzymes for the UvrC protein. In support of this hypothesis, there is strong relationship between the presence of *cho *genes and the absence of these endonuclease genes in several bacterial genomes, as it is shown in Table 3. But whether these small endonucleases share with the Cho protein similar mechanisms for incision, and which lesions they recognize, remain open questions. In Figure 1, a scheme representing the UvrC and the UvrC-like proteins illustrates their different domains. Although these putative endonucleases share low similarity with the larger Cho protein, they all contain a similar domain (COG2827). It is important to mention that one of these UvrC-like genes in *C. crescentus *(CC3518) presents an SOS box, indicating that it is under control of the LexA repressor control and is part of the SOS regulon [21]. This reinforces the hypothesis that this protein acts in DNA repair mechanisms.

Transcription-coupled repair is most likely to be functional in the *C. crescentus *genome, since the gene *mfd *and the complete NER pathway are found in this genome. Mfd is a well conserved bacterial protein which is a coupling factor for NER with transcription. In *E. coli *the enzyme encoded by this gene is capable of removing the transcription complex when it is found stalled on damage, leaving room for DNA excision repair recruitment to the site of a DNA lesion [31].

#### C – Mismatch repair (MMR)

The MMR pathway acts as an editor, correcting mismatched base pairs introduced in DNA by several processes, including replication and recombination. The complex MutS/MutL recognizes a DNA replicative error or misalignment, followed by excision of the section containing the mismatch [32]. In *C. crescentus*, MMR is probably performed by *mutS*/*mutL *homologs (Table 4). In *E. coli*, MutH is an endonuclease that participates in the recognition of GATC methylated sequences, discriminating the DNA strand to be repaired by MMR. In *C. crescentus*, there is no *mutH *homolog, which correlates well with the absence of *dam *homologs that normally perform GATC methylation. However, it should be remarked that *mutH *homologs are rarely found in other bacterial genomes. Therefore, this implies that *C. crescentus *and other bacteria must use different proteins for strand recognition and incision to complete MMR.

Very short patch (Vsp) repair is initiated by the action of the protein Vsr, which is also an endonuclease. This pathway corrects T:G mismatches to C:G base pairs, if this mismatch is within a hemi-methylated Vsr recognition site. The endonuclease activity of Vsr, responsible for cutting 5' of the misplaced T, is stimulated by MutL in *E. coli *[33]. An ortholog of Vsr is found in the *C. crescentus *genome, but its function remains to be confirmed.

### 2 – Direct repair

Several repair mechanisms revert lesions in DNA, removing damage by a single step. The reversion can be achieved by photoreactivation, alkyltransfer and oxidative demethylation. The corresponding genes found in *C. crescentus *are shown in Table 5.

#### A – Photoreactivation

**Figure 2:**
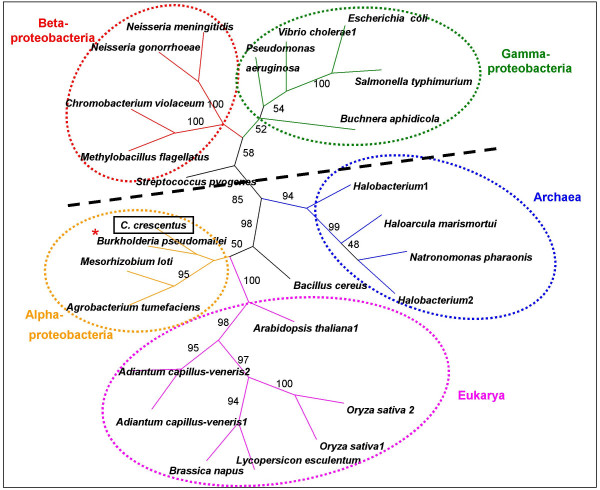
Consensus unrooted tree generated by the Neighbor-Joining distance method for the Phr protein. The symbol * indicates the beta proteobacteria in a different bacterial group. The circles highlight the main groups of Bacteria, Archaea and Eukarya. The homolog of *C. crescentus *is indicated inside the square box.

The mechanism of photoreactivation is performed by enzymes known as photolyases, which employ visible light as energy source to monomerize pyrimidine dimers induced by UV irradiation. The photolyase family includes repair proteins (photolyases) and blue light receptors (cryptochromes), the common feature of these proteins being the presence of a FAD chromophore as cofactor. These genes are well conserved and are found throughout the three domains of life, from bacteria to aplacental mammals [34]. Only one photolyase is found in *E. coli*, while *C. crescentus *has two putative homologs of this family (Table 5), although one of these is only photolyase-related, and its function in DNA repair is not clear. A phylogenetic tree for the main Phr proteins is presented in figure 2. The photolyase-related gene was not included as it is highly divergent. Curiously, the *C. crescentus *protein is located far from the known bacterial photolyases and closer to the branch where the Eukaryotes (mainly plants) are found. On the other hand, some recently described *phr *bacterial orthologs (from another alpha-proteobacteria and some firmicutes) are also in the same branch. It is difficult to establish the origin of such a gene, as it may have occurred from an old duplication later lost in most of the bacterial species. Alternatively, this could represent a potential horizontal gene transfer event involving Eukarya and Bacteria. Tree topology also indicates that the *C. crescentus phr *gene may not be involved in DNA damage repair, as their known orthologs of plants are *Cry1 *genes, which are blue light photoreceptors related only to the regulation and development of plant growth [35]. Although this phylogenetic approach has been very useful for gene function predictions [36], the participation of the *C. crescentus phr *gene in photorepair must be determined experimentally.

#### B – Alkyltransferase

**Figure 3:**
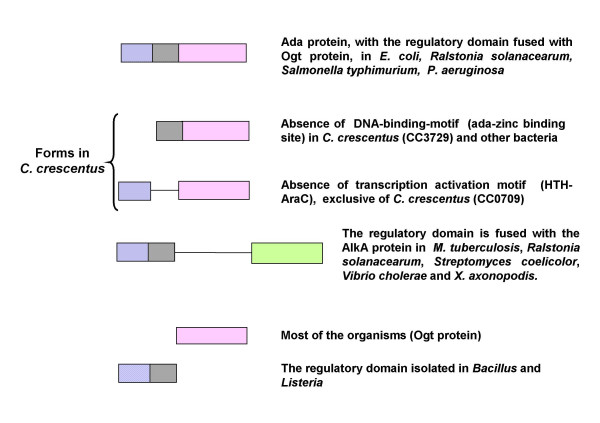
**Representation of the regulatory domain patterns, the alkyltransferase of the Ada, and the Ogt proteins in Bacteria.** The regulatory portion of ada is represented by blue and gray boxes, respectively the DNA binding (ada-zinc binding site) and transcriptional activation (HTH-AraC) domains. The alkyltransferase domain, also present in the Ogt protein, is represented by pink boxes. The green box characterizes the AlkA domain, which is involved in the removal of alkyl groups in the base excision repair pathway. The AlkA protein is fused to the regulatory portion of Ada protein in some organisms, as shown.

Proteins participating in this mechanism, products of the *ada *and *ogt *genes, are alkyltransferases which transfer alkyl adducts from damaged DNA to themselves, in a non-enzymatic suicide mechanism [37]. Ada is also an important and interesting protein involved in gene expression regulation. The protein is normally comprised of a N-terminal regulatory domain, and a C-terminal catalytic domain, but other bacteria present other forms of gene organization (Figure 3). In *E. coli *the regulatory and alkyltransferase functions are present in the product of a unique gene (*ada*). Homologs to the alkyltransferase domain (normally called Ogt, for O_6_-alkyl-guanine transferase) also occur independently of the regulatory Ada domain, and are present and highly conserved in several organisms of the three domains of life [38]. Thus, this may be an ancient protein, and the Ada protein is probably a result of domain fusion. The regulatory domain also appears fused with an alkyl glycosylase domain in some bacteria. This domain, related to the AlkA protein of *E. coli*, is also important in the process of removing alkylated bases from DNA (BER). Interestingly, the *Ralstonia *genome presents genes that encode for both combinations of Ada proteins (Ada-Ogt and Ada-AlkA). In other organisms such as *Bacillus subtilis *[39] and *Listeria*, two different genes encode each domain separately, thus reinforcing the idea of gene fusion as the origin of Ada organization. While in *Bacillus subtilis *the two domains overlap by 11 base pairs, those in *Listeria *are arranged in the opposite direction.

In the *C. crescentus *genome there are four orthologs of the alkyl transferase gene, two that do not present any N-terminal regulatory domain (*ogt *orthologs), and two that have the regulatory domain similar to the *E. coli ada *gene (Table 5). The presence of these four genes reinforces the importance of this type of repair for this free-living bacterium. However, the two *ada *orthologs of *C. crescentus *are different as regards the regulatory domain, not previously described. The commonly found regulatory *ada *domain found in nature comprises two different protein motifs: one related to DNA binding (ada-zinc binding site) [40] and one potentially responsible for a transcriptional activation (HTH-AraC) [41]. In the two *C. crescentus ada *orthologs, the N-terminus lacks either one or the other of the portion motifs. While homologs of the gene lacking DNA-binding-motif (CC3729) are found in many other bacteria, the copy lacking the transcription activation motif (CC0709) is still unique for the *C. crescentus *genome. The meaning and role of such different alkyl transferase orthologs in this bacterium deserve further experimental investigation.

#### C – Oxidative demethylation

Alkylated damage may also be repaired through oxidative demethylation [10]. In *E. coli*, the single gene participating in this pathway is *alkB*, whose transcription is under *ada *gene regulation. This protein is well conserved with homologs in various organisms, including human beings. The protein removes the methyl group from 1-methyladenine and 3-methyl-cytosine, by an alpha-ketoglutarate dependent oxidative reaction, releasing formaldehyde, and healing the damage [10]. An *alkB *ortholog is found in the *C. crescentus *genome, and the phenotype of a mutant strain confirms hypersensitivity to the alkylating agent MMS [8]. These results validate the role of the *alkB *ortholog for the repair of alkyl DNA lesions in *C. crescentus*.

### 3 – Recombinational repair

Recombinational repair is crucial in maintaining genome integrity, since it is necessary for repairing single-strand gaps and double-strand DNA breaks or restoring the replication fork. In *E. coli*, there are two pathways that act independently of each other to initiate recombinational repair. The so-called RecBCD and RecFOR pathways recruit RecA to single-stranded DNA, to initiate the repair of double strand breaks or of post-replication daughter-strand gap, respectively [42]. The RecBCD pathway is absent in *C. crescentus *(Table 6), but this may be replaced by a functionally equivalent pathway, AddAB, as it has been shown for another alpha proteobacterium, *Rhizobium etli *[43]. The RecBCD function can also be provided, in part, by RecFOR, as has been suggested for *D. radiodurans *[44] and for *E. coli*, where the viability of RecBCD mutants is restored by the RecFOR pathway [45]. On the other hand, the absence of the complete SbcBCD anti-recombination pathway may be related to the absence of RecBCD, as this is also observed for genomes from other alpha-proteobacteria and mollicutes [46].

The initial step of recombination is followed by the resolution of the Holliday junction by helicase and endonuclease activities, performed by members of RuvABC, a pathway that is complete in *C. crescentus*. The presence of another gene in *C. crescentus *(CC1283), an ATPase related to *ruvB *subunit, the only redundancy present in the recombinational repair pathway in this bacterium, should be mentioned. The lack of homologs of *recE *and *recT *genes is not surprising as these are encoded by a prophage being restricted to some *E. coli *K12-strains. Moreover, genes involved in the non-homologous end-joining pathway of DNA repair (similar to the Ku proteins of Eukaryotes) that have been identified in a number of bacterial species [47] were not found in *C. crescentus*.

### 4 – Other repair related proteins

#### A – SOS system

The SOS regulon is a set of physiological responses to damage in DNA. In *E. coli*, this is well described and involves the participation of more than 40 genes [48]. Under normal conditions, the LexA protein curbs the expression of SOS genes by binding to their promoters. The induction of such a system stems from the coprotease activity of the protein RecA, which inactivates the LexA repressor. The presence of the main regulatory genes of the SOS response (*lexA *and *recA*) in *C. crescentus *(Table 7) indicates a similar SOS regulon. However, the number of genes under SOS control remains to be investigated in *C. crescentus*. The SOS functions are normally induced in response to stress conditions to ensure survival, but the mutation rates can be increased. The genes *umu*C and *umu*D form a complex UmuC/UmuD_2_, known as DNA polymerase V [49], being responsible to a large degree for the induced mutagenesis by the SOS system in *E. coli*, but this polymerase is absent in *C. crescentus*. It is intriguing that the SOS inducible and error prone DNA polymerase IV (*din*B), also involved in translesion synthesis in *E. coli *[50], presents a different regulation in *C. crescentus*. In fact, the SOS induced mutagenesis in *C. crescentus *has been shown to be promoted by enzymes encoded by an operon, including a second copy of DnaE (the catalytic subunit of DNA polymerase III) and other genes named *imuA *and *imuB *[21]. Orthologs to *imuAB *and *dnaE2 *genes are found in many other genomes, indicating that this enzymatic machinery is widely implicated in stress induced mutagenesis and contributes to genetic variability in the bacterial domain.

The nudix-hydrolase superfamily includes the *E. coli *MutT, which hydrolyses 8-oxodGTP, avoiding its incorporation during DNA synthesis, and other genes that may also be involved in the sanitization of the nucleotide pool [51]. The existence of several proteins of this family in bacterial genomes is quite common, and, for example, in *D. radiodurans *22 proteins of such family have been identified [52]. In *C. crescentus*, we have found 11 proteins (Table 7) that share the NUDIX motif, but CC0833 is most probably the *mutT *ortholog.

The *recX *gene, which codes for a negative regulator of the RecA function in *E. coli*, has been detected in most bacterial species [53,54]. However, RecX homologs were not found in *C. crescentus *or in other alpha proteobacteria, suggesting a different type of RecA regulation for this group of bacteria.

#### B – Ligases

The DNA ligases are essential enzymes in the process of repair, replication and recombination, joining the recently synthesized DNA to the intact strand of DNA. They comprise two families: the ATP-dependent (type II family) ligases are characteristic of Eukaryotes and Archaea, and the NAD-dependent (type I family) ligases are characteristic of Bacteria. Sometimes, both families are present in bacteria, as is the case of *C. crescentus *(Table 7). The participation of the ATP-dependent ligase in DNA recombination and repair has been proposed [55].

#### C – Helicases

The helicases change the structure of DNA and RNA to allow for the access to transcriptional machinery. The gene *lhr *encodes a large helicase. The restricted distribution of this helicase in bacteria and its common duplication among the Archaea suggest that the gene may be a recent acquisition, through horizontal gene transfer, by some bacteria [56]. In *C. crescentus*, we have identified one copy of this gene as is shown in Table 7. Another interesting DNA-dependent ATPase and helicase is the DinG protein, and a homolog was found in *C. crescentus *(CC2038) [57]. This protein is a bacterial homolog for human helicases that are known to participate in DNA repair, and mutations have been related to some human genetic diseases.

Conclusion

As very little is known about DNA repair in *C. crescentus*, the relevance of this comparative analysis is to provide the basis for investigating the putative genes and pathways detected in the genome of this bacterium. Many of the predictions generated through bioinformatic analyses also contributed to the identification of many gene duplications and modifications, which raised potentially different strategies of DNA repair in *C. crescentus *and other related bacteria.

DNA repair genes represent the core DNA metabolism, are in general strongly conserved, and *C. crescentus *has, as expected, many similarities to *E. coli*. However, the absence of some orthologs may reflect components of proteins that can be backed up by other repair pathways, as for the RecBCD complex. Otherwise, the main genes related to BER and NER pathways, including transcription-coupled repair, are detected in *C. crescentus*, as well as many of the recombinational repair genes. As in *E. coli*, an SOS regulon is found in *C. crescentus*, since the main genes (*recA *and *lexA *repressor) are identified, although SOS induced mutagenesis is related to a different mechanism [21]. *C. crescentus *also has some interesting non-identical gene duplications which include DNA ligases, the main subunit (DnaE) of DNA polymerase III, exonuclease III and alkytransferases. Interesting gene structures were identified for the alkyltransferases, one of which is still unique for *C. crescentus*.

Although most of the *in silico *inferences must be confirmed and tested by experimentation, this work provides a profile of those genes responsible for the maintenance of genome stability in *C. crescentus*, contributing to the understanding of the mechanisms of genome protection and mutagenesis in alpha-proteobacteria. It also provides a useful framework for further investigations on the functions of these genes.

## Methods

### Identification of DNA repair genes

The putative ORFs in *C. crescentus *and *E. coli *were compared with known DNA repair related genes in public databases using the BlastP search in Genebank non-redundant (nr) database [58]. In some specific cases, potential DNA repair genes in *C. crescentus *genome (GenBank accession n° AE005673) were identified both by sequence similarity searches (using as seed sequences orthologs from other organisms) and keyword searches. *C. crescentus *candidate genes were thus confirmed both by sequence similarity searches (BlastP program) and domain analysis.

### Phylogenetic analyses

Phylogenetic trees were generated for a group of Phr protein homologs. Protein sequences were aligned using the ClustalX multiple sequence alignment program [59] with manual adjustment with Genedoc (v2.6.02). Only unambiguously aligned positions (excluding poorly conserved and gap regions) were used in phylogenetic analysis, which was performed using the Phylip program version 3.5 [60]. Parsimony analysis was conducted using the Protpars program, whereas distance methods were performed using the Neighbor-Joining [61] method in Phylip. The distance matrix was constructed using a PAM matrix model [62]. Bootstrap support (resampled 1,000 times) was calculated, and strict consensus trees constructed. Only bootstrap values greater than 50% are shown. Similar topologies were found for both algorithms employed, only Neighbor-Joining being displayed. The consensus trees obtained were viewed through TreeView software [63]. In this work, the option for non-rooted trees aims at demonstrating only the relationship among organisms, without, however, linking ancestors and descendants. Organism names with the accession code of the Phr and Phr-like proteins analyzed are shown in Table 8.

## Authors' contributions

MM-P performed gene search and functional classification, and together with CFMM, designed, conceived the ideas and the writing of the manuscript. RCPM helped on gene analysis. All authors read and approved the final manuscript.

## Figures and Tables

**Table 1:** Putative DNA repair genes of *C. crescentus *for Base Excision Repair

	**Genes in *E. coli *(protein size)**	**Description**	**Orthologs in *C. crescentus***	**Similarity with *E. coli***^***a***^
**Monofunctional DNA glycosylases**	*alkA *(282 aa)	3-methyl-adenine DNA glycosylase II, inducible enzyme	CC2201	74/168 (44%)
	*mutY *(350 aa)	adenine glycosylase	CC0377	178/350 (50%)
	*tag *(187 aa)	3-methyl-adenine DNA glycosylase I, constitutive enzyme	CC0382	116/179 (64%)
	*ung *or *udg *(229 aa)	uracil-DNA-glycosylase	1-CC2084; 2-CC1333; 3-CC2333; 4-CC1549;	**no similarity**

**Bifunctional DNA glycosylases**	*fpg (mutM) *(269 aa)	formamidopyrimidine DNA glycosylase	CC3707	150/286 (52%)
	*nei *(263 aa)	endonuclease VIII and DNA N-glycosylase with an AP lyase activity	**absent**	
	*nth *(211 aa)	endonuclease III; specific for apurinic and/or apyrimidinic sites	1-CC3731; 2-CC2272	136/204 (66%) 77/190 (40%)

**AP endonucleases**	*nfo *(285 aa)	endonuclease IV	**absent**	
	*nfi *(223 aa)	endonuclease V	**absent**	
	*xthA *(268 aa)	exodeoxyribonuclease III	1-CC3706 2-CC2011;	140/282 (49%) 138/274 (50%)

a. numbers indicate similarity within local alignment in aminoacids (aa).

**Table 2:** Genes encoding putative DNA repair proteins for Nucleotide Excision Repair

**Genes in *E. coli *(protein size)**	**Description**	**Orthologs in *C. crescentus***	**Similarity with *E. coli***^***a***^
*mfd *(1148 aa)	transcription-repair coupling factor	CC1844	641/1113 (57%)
*uvrA *(940 aa)	DNA damage recognition protein UvrA	CC2590	716/944 (75%)
*uvrB *(673 aa)	DNA damage binding protein UvrB	CC2981	481/668 (72%)
*uvrC *(610 aa)	excision nuclease UvrC	1-CC2881;	360/608 (59%)
	UvrC-N-terminal like protein, endonuclease	2-CC3518;	**absent**
		3-CC1343;	**absent**
		4-CC0707	**absent**
*uvrD *(720 aa)	DNA-dependent ATPase I and helicase II	CC1528	373/636 (58%)

a. numbers indicate similarity within local alignment in aminoacids (aa).

**Table 3:** Distribution of UvrC and UvrC-like proteins in different bacterial genomes.

**Organisms**	**Group**	**UvrC**	**Endonuclease**	**Cho protein**	**Fusion Cho-polIII**
***Caulobacter crescentus***	Alpha-proteobacteria	+	+		
***Mesorhizobium loti***	Alpha-proteobacteria	+	+		
***Rhodopseudomonas palustris***.	Alpha-proteobacteria	+	+		
***Azoarcus sp***	Beta-proteobacteria	+			+
***Chromobacterium violaceum***	Beta-proteobacteria	+			+
***Dechloromonas aromática***	Beta-proteobacteria	+			+
***Methylobacillus flagellatus***	Beta-proteobacteria	+			+
***Neisseria meningitidis***	Beta-proteobacteria	+	+		
***Nitrosospira multiformis***	Beta-proteobacteria	+			+
***Ralstonia eutropha***	Beta-proteobacteria	+			+
***Ralstonia metallidurans***	Beta-proteobacteria	+			+
***Rhodoferax ferrireducens***	Beta-proteobacteria	+			+
***Thiobacillus denitrificans***	Beta-proteobacteria	+			+
***Alkaliphilus metalliredigenes***	Firmicutes	+	+		
***Mycobacterium tuberculosis***	Firmicutes	+			+
***Flavobacterium sp***	Flavobacteria	+	+		+
***Azotobacter vinelandii***	Gamma-proteobacteria	+		+	
***Escherichia coli***	Gamma-proteobacteria	+		+	
***Legionella pneumophila***	Gamma-proteobacteria	+	+		
***Salmonella sp***.	Gamma-proteobacteria	+		+	
***Shiguella flexneri***	Gamma-proteobacteria	+		+	
***Xanthomonas sp***.	Gamma- proteobacteria	+		+	

**Table 4:** *C. crescentus *genes identified for Mismatch Repair pathway

**Genes in *E. coli *(protein size)**	**Description**	**Orthologs in *C. crescentus***	**Similarity with *E. coli***^***a***^
*dam *(278 aa)	Methylase GATC	**absent**	
*mutH *(229 aa)	Endonuclease GATC	**absent**	
*mutL *(615 aa)	DNA mismatch repair protein	CC0695	188/331 (56%)
*mutS *(853 aa)	DNA mismatch repair protein	CC0012	461/842 (55%)
*vsr *(156 aa)	Very short patch repair protein	CC3624	74/127 (58%)

a. numbers indicate similarity within local alignment in aminoacids (aa).

**Table 5:** *C. crescentus *genes identified for Direct Repair pathway

**Genes in *E. coli *(protein size)**	**Description**	**Orthologs in *C. crescentus***	**Similarity with *E. coli***^***a***^
*ada *(354 aa)	O6-methylguanine-DNA methyltransferase; transcription activator/repressor enzyme	1-CC0709 (with internal deletion);	**absent**
		2-CC3729	139/295 (47%)
*alkB *(216 aa)	AlkB protein, 1-methyladenine and 3-methylcytosine repair protein	CC0009	107/186 (57%)
*phr *(472 aa)	Photolyase	1-CC1428	260/474 (54%)
	Photolyase-like	2-CC0646	**absent**
*ogt *(171 aa)	O-6-alkylguanine-DNA/cysteine-protein methyltransferase	1-CC0689;	95/166 (57%)
		2-CC0659	53/106 (50%)

a. numbers indicate similarity within local alignment in aminoacids (aa).

**Table 6:** Putative recombinational repair genes of *C. crescentus*.

**Genes in *E. coli***	**Description**	**Genes in *C. crescentus***	**Similarity with *E. coli***^***a***^
**absent**	ATP-dependent helicase,/nuclease (*addA*)	CC3538	
**absent**	ATP-dependent nuclease (*addB*)	CC3537	
*radA * (460 aa)	DNA repair protein RadA	CC1660	280/456 (61%)
*recA * (353 aa)	DNA strand exchange and renaturation, DNA-dependent ATPase, DNA- and ATP-dependent coprotease	CC1087	257/321 (80%)
*recB * (1180 aa)	DNA helicase, ATP-dependent dsDNA/ssDNA exonuclease V subunit, ssDNA endonuclease	**absent**	
*recC * (1122 aa)	DNA helicase, ATP-dependent dsDNA/ssDNA exonuclease V subunit, ssDNA endonuclease	**absent**	
*recD * (608 aa)	DNA helicase, ATP-dependent dsDNA/ssDNA exonuclease V subunit, ssDNA endonuclease	**absent**	
*recE * (866 aa)	exonuclease VIII, ds DNA exonuclease, 5' → 3' specific	**absent**	
*recF * (357 aa)	Recombinational repair protein	CC0159	129/272 (47%)
*recG * (693 aa)	DNA helicase, resolution of Holliday junctions, branch migration	CC1437	362/671 (53%)
*recJ * (577 aa)	ssDNA exonuclease, 5' → 3' specific	CC1386	281/551 (51%)
*recN * (553 aa)	protein used in recombination and DNA repair	CC1983	290/570 (51%)
*recO * (242 aa)	Bacterial Recombinational repair protein	CC1564	79/165 (47%)
*recQ * (609 aa)	ATP-dependent DNA helicase	CC3465	337/604 (55%)
*recR * (201 aa)	recombination and repair putative	CC0269	109/192 (56%)
*recT * (269 aa)	recombinase, DNA renaturation	**absent**	
*recX * (166 aa)	RecA regulator RecX	**absent**	
*rus * (120 aa)	endodeoxyribonuclease Rus (Holliday junction resolvase)	**absent**	
*ruvA * (203 aa)	Holliday junction helicase subunit A; branch migration	CC3237	98/204 (48%)
*ruvB * (336 aa)	Holliday junction helicase subunit B; branch migration	1-CC3236;	250/318 (78%)
		2-CC1283	67/136* (49%)
*ruvC * (173 aa)	Holliday junction nuclease; resolution of structures	CC3238	85/151 (56%)
*sbcB * (475 aa)	exodeoxyribonuclease I	**absent**	
*sbcC * (1048 aa)	ATP-dependent dsDNA exonuclease	**absent**	
*sbcD * (400 aa)	ATP-dependent dsDNA exonuclease	**absent**	
*ssb * (163 aa)	ssDNA-binding protein factor	CC1468	84/110 (76%)

a. numbers indicate similarity within local alignment in aminoacids (aa). * These genes have coverage lower than 50%.

**Table 7:** Other repair related proteins in *C. crescentus*

**Genes in *E. coli***	**Description**	**Orthologs in *C. crescentus***	**Similarity with *E. coli***^***a***^
*dcm *(472 aa)	DNA-cytosine methyltransferase	CC3626	158/395 (40%)
*dinG *(716 aa)	probably ATP-dependent helicase	CC2038	213/479 (44%)
*dinB *(pol IV) (351 aa)	Polymerase IV (damage-inducible protein B)	CC2466	185/320 (57%)
*dnaE *(1160 aa)	subunit of DNA polymerase III	1- CC1926; 2-CC3211	659/1121 (58%) 503/1090 (46%)
*dut *(151 aa)	dUTPase	CC3713	86/136 (63%)
*lexA *(202 aa)	regulator for SOS regulon	CC1902	110/205 (54%)
*lhr *(1538 aa)	Lhr-like helicases	CC2040	373/973 (41%)
*lig *(671 aa)	DNA ligase	1-CC1522 NAD-dependent 2-CC3610 ATP-dependent	427/678 (63%) **absent**
*mutT *(129 aa)	mutT/NUDIX family	1-CC0833 2-CC3382 3-CC0407 4-CC0418 5-CC1554 6-CC1126 7-CC3440 8-CC1346 9-CC3650 10-CC0266 11-CC2652	69/124 (55%) 47/91 (51%) 38/64* (59%) 32/60* (53%) 41/95 (43%) 33/69 (47%) 46/102 (45%) 27/56* (48%) 28/59* (47%) 22/45* (48%) 17/23* (73%)
*polB *(783aa)	DNA polymerase II	**absent**	
*radC *(222 aa)	DNA repair protein	1-CC2680 2-CC2744	101/172 (58%) 38/56* (67%)
*umuC/umuD*	pol V, Y-family DNA polymerase	**absent**	
**absent**	*imuA*	CC3213	
**absent**	*imuB*, Y-family DNA polymerase	CC3212	

a. numbers indicate similarity within local alignment in aminoacids (aa). * These genes have coverage lower than 50%.

**Table 8:** Genes of DNA photolyase/cryptochrome protein family investigated in the phylogenetic analysis. The organisms are listed in alphabetic order within the taxa.

**Organisms (Abbreviation)**	**Division**	**Acession number**
***Haloarcula marismortui *ATCC 43049**	Archaea	gi|55377670
***Halobacterium sp***	Archaea.	gi|10581208; gi|10580849
***Natronomonas pharaonis DSM 2160***	Archaea	gi|76802655
***Bacillus cereus ATCC 10987***	Firmicutes	gi|42738164
***Streptococcus pyogenes ***	Firmicutes	gi|13622594
***Agrobacterium tumefaciens str. C58***	Alpha- proteobacteria	gi|17935123
***Caulobacter crescentus ***	Alpha- proteobacteria	gi|13422791
***Mesorhizobium loti ***	Alpha -proteobacteria	gi|14027464
***Burkholderia pseudomallei***	Beta-proteobacteria	gi|76578109
***Chromobacterium violaceum***	Beta-proteobacteria	gi|34498936
***Methylobacillus flagellatus***	Beta-proteobacteria	gi|91708933
***Neisseria meningitidis***	Beta-proteobacteria	gi|7380817
***Neisseria gonorrhoeae***	Beta-proteobacteria	gi|59802028
***Buchnera aphidicola***	Gamma-proteobacteria	gi|11133758
***Escherichia coli***.	Gamma-proteobacteria	gi|16128683
***Pseudomonas aeruginosa ***	Gamma-proteobacteria	gi|15599855
***Salmonella typhimurium LT2***	Gamma-proteobacteria	gi|16419217
***Vibrio cholerae***	Gamma-proteobacteria	gi|9657440
***Brassica napus ***	Eukaryote	gi|94965681
***Lycopersicon esculentum ***	Eukaryote	gi|8101442
***Oryza sativa***	Eukaryote	gi|28201254; gi|21740729
***Adiantum capillus-veneris***	Eukaryote	gi|3551225; gi|3551223
***Arabidopsis thaliana***	Eukaryote	gi|30678870
